# Zinc Enrichment in Two Contrasting Genotypes of *Triticum aestivum* L. Grains: Interactions between Edaphic Conditions and Foliar Fertilizers

**DOI:** 10.3390/plants10020204

**Published:** 2021-01-21

**Authors:** Inês Carmo Luís, Fernando C. Lidon, Cláudia Campos Pessoa, Ana Coelho Marques, Ana Rita F. Coelho, Manuela Simões, Manuel Patanita, José Dôres, José C. Ramalho, Maria Manuela Silva, Ana Sofia Almeida, Isabel P. Pais, Maria Fernanda Pessoa, Fernando Henrique Reboredo, Paulo Legoinha, Mauro Guerra, Roberta G. Leitão, Paula Scotti Campos

**Affiliations:** 1 Earth Sciences Department, Faculdade de Ciências e Tecnologia, Campus da Caparica, Universidade Nova de Lisboa, 2829-516 Caparica, Portugal; fjl@fct.unl.pt (F.C.L.); c.pessoa@campus.fct.unl.pt (C.C.P.); amc.marques@campus.fct.unl.pt (A.C.M.); arf.coelho@campus.fct.unl.pt (A.R.F.C.); mmsr@fct.unl.pt (M.S.); mfgp@fct.unl.pt (M.F.P.); fhr@fct.unl.pt (F.H.R.); pal@fct.unl.pt (P.L.); 2 GeoBioTec Research Center, Faculdade de Ciências e Tecnologia, Campus da Caparica, Universidade Nova de Lisboa, 2829-516 Caparica, Portugal; mpatanita@ipbeja.pt (M.P.); cochichor@mail.telepac.pt (J.C.R.); abreusilva.manuela@gmail.com (M.M.S.); sofia.almeida@iniav.pt (A.S.A.); isabel.pais@iniav.pt (I.P.P.); paula.scotti@iniav.pt (P.S.C.); 3 Escola Superior Agrária, Instituto Politécnico de Beja, R. Pedro Soares S/N, 7800-295 Beja, Portugal; jdores@ipbeja.pt; 4 PlantStress & Biodiversity Lab, Centro de Estudos Florestais (CEF), Instituto Superior Agronomia (ISA), Universidade de Lisboa (ULisboa), Quinta do Marquês, Av. República, 2784-505 Oeiras, Portugal; 5 ESEAG-COFAC, Avenida do Campo Grande 376, 1749-024 Lisboa, Portugal; 6 Instituto Nacional de Investigação Agrária e Veterinária, I.P. (INIAV), Estrada de Gil Vaz 6, 7351-901 Elvas, Portugal; 7 Instituto Nacional de Investigação Agrária e Veterinária, I.P. (INIAV), Avenida da República, Quinta do Marquês, 2780-157 Oeiras, Portugal; 8 LIBPhys-UNL, Physics Department, Faculdade de Ciências e Tecnologia, Campus da Caparica, Universidade Nova de Lisboa, 2829-516 Caparica, Portugal; mguerra@fct.unl.pt (M.G.); rg.leitao@fct.unl.pt (R.G.L.)

**Keywords:** agronomic biofortification, bread wheat, grain yield, zinc foliar application, zinc grain content

## Abstract

This study aimed to assess the implications of Zn enrichment in wheat grains as a function of contrasting genotypes, edaphic conditions and foliar fertilizers. *Triticum aestivum* L. varieties Roxo and Paiva were grown in four production fields, and sprayed with ZnSO_4_ (0, 16.20 and 36.40 kg/ha) Zn-EDTA (0, 6.30 and 12.60 kg/ha) and Tecnifol Zinc (0, 3.90 and 7.80 kg/ha). The heterogeneous edaphic conditions of the wheat fields were chemically characterized, it being found that soil properties determine different Zn accumulation in the grains of both genotypes. Foliar spraying enhanced to different extents Zn content in the grains of both genotypes, but the average of enrichment indexes varied among the wheat fields. Zinc mostly accumulated in the embryo and vascular bundle and to a lesser extent in the endosperm. Grain yield and test weight sprayed by ZnSO_4_ gave the highest values in both genotypes, but the opposite was found for Zn-EDTA. Considering the color parameters, lightness and red–green transitions were found to be a conjunction of genotype characteristics, fertilization types and edaphic conditions prevailing in each field. It is concluded that the index of Zn enrichment in wheat grains is a docket of edaphic conditions, genotype and type of fertilization.

## 1. Introduction

By 2050, several estimates suggest that the world population will reach about 9.7 billion people, emphasizing huge disparities between developed and developing countries [1,2], which creates an urgent need for an increasing production of staple foods. Besides, the concentration and bioavailability of micronutrients required for the human diet have decreased in staple foods [3]. In fact, nowadays about 3 billion people suffer from malnutrition, approximately 2 billion have micronutrients deficiencies, 1.9 billion are obese or overweight and 821 million are undernourished [4,5]. Malnutrition also affects about one in three people, contributing to 45% of child deaths and major risks of contracting infectious diseases, wasting and/or stunting, reduced early child cognitive development and developing chronic diseases that are not contagious [5,6]. Micronutrient deficiencies in developing countries further diminish work productivity and increase healthcare costs, leading these countries to great economic losses [3,7,8]. Additionally, although the recommended dietary allowance (RDA) of Zn for adults is, approximately, 8 mg/day for women and 11 mg/day for men [9], poor Zn intake, high loss and low solubility determines Zn deficiency [10], leading to a weakening of the immune system, loss of brain function and changes in physical growth [4,10]. Additionally, there are some other diseases and health conditions associated with Zn deficiency such as cancer, risk of infections, infertility, problems regarding learning skills and mental lethargy. Nevertheless, Zn excess in a diet might also cause some diseases, namely, altered lymphocyte function, epigastria pain and nausea [11].

A poor diet in micronutrients can lead to physiological lacks, especially if consumers ingest plant-derived foods. This applies not only to the consumption of edible plant parts with a low concentration of micronutrients, but also to plant growth in soils with poor nutrient availability that can culminate in nutrient deficiencies in the world population [3,6,12]. In this context, in 2020, the world production of wheat will probably reach 762.6 million tons [13] but will only keep an average consumption of 67.5 kg per person a year [12,14]. Therefore, human dependence upon cereals with a poor Zn status deepens the gap between the available amount and the amount required for good health, which is 40–50 mg/kg [15]. Indeed, although in plants the normal Zn concentration might range between 25 and 150 mg/kg, this nutrient content is lower in bread wheat grains (varying between 20–35 mg/kg, with an average value ranging between 28–30 mg/kg) [7]. Zinc has a large number of functions such as playing an important role in plant growth, namely as a co-factor in the auxin metabolism, enzymatic activation and synthesis of chlorophyll and nucleotides [7,16]. Likewise, Zn is also important in the expression and regulation of genes [17] and Zn transport from roots to the xylem can be apoplastic, but the symplastic circulation prevails [18]. In the xylem, Zn flows in its ionic form (Zn^2+^) or complexed with histidine and nicotinamide, occurring complexed with organic acids in the chloroplast [19,20,21]. The Zn^2+^ is further transported to the leaves and is moved in the phloem by ZIPs and YSL proteins, and it can be mediated by ZIPs (ZIP1, ZIP3 and ZIP4), or by Ca^2+^ channels in plasmatic membranes [11,22]. Bread wheat, as a staple food, can be considered a good target for Zn biofortification (i.e., for enrichment of the content or the nutrient bioavailability in the edible parts of staple crops during plant growth) [7,23,24,25,26]. From an agronomic perspective, the crucial time to apply Zn to wheat crops is during the grain filling [7], due to the intense flow of nutrients from vegetative organs to the grains, such as the root capture of Zn from soils, its translocation to the shoots and further remobilization of stored nutrients in leaves [21]. Besides, the application of adequate NPK fertilizers can further optimize the effects of foliar and soil applications of Zn [7]. Yet, more than 30% of the world soils present Zn deficiencies [11] and its concentration, generally, ranges between 6–28 mg/kg [8]. Besides, different levels of Zn deficiencies in soils (particularly calcareous soils) trigger distinct susceptibility in crops (namely its strong deficiency) and its application in soils is less efficient than foliar spraying when it comes to grain accumulation [6,11]. Nevertheless, soil fertilization with Zn promotes a high grain yield [11,27], but a combined application of soil and foliar spraying further results in an increased grain yield and Zn accumulation [28]. In this context, through foliar spraying, the uptake of inorganic nutrients, namely Zn, requires this nutrient movement/absorption across the cuticle (through a dissolution–diffusion process) and/or through the stomatal cavity [29,30]). Yet, the foliar uptake kinetics are influenced by the characteristics of the leaf surface, including the thickness of the wax layer and the distribution of stomata and trichomes [31,32,33]. Thereafter, the overall efficacy of Zn movement is linked to its subsequent loading into the foliar vascular systems and translocation via the phloem of primary veins into the other plant tissues [30,34,35,36]. Zn fertilization, through soil or foliar application, therefore is an effective method for improving this nutrient concentration in the grain [15]. Following this assumption, this study aimed to develop an itinerary for Zn enrichment of *Triticum aestivum* L. grains, using as test systems Roxo and Paiva Portuguese varieties sprayed with ZnSO_4_, Zn-EDTA and Tecnifol Zinc (a highly soluble Zn mixture having Zinc sulphate mono, hexa and heptahydrate). As heterogeneous properties prevail in the soils, both wheat genotypes were Zn biofortified in four experimental fields, with Zn accumulation and tissue localization, the yield, test weight, thousand kernel weight, ashes and colorimetric properties being assessed.

## 2. Results

### 2.1. Soil Analysis

The experimental fields 1 and 2 showed (Table 1) have a higher electrochemical conductivity than fields 3 and 4 (in µS/cm, 507.44/521.78 and 368.56/328.00, respectively). The pH showed a similar trend, with fields 3 and 4 revealing the same value (7.67/7.80 and 6.85, respectively). The organic matter did not vary significantly in fields 1, 2 and 3, but lower values were found in field 4 (to ca. 73.6%, relatively to field 3). Moisture displayed the highest value in field 1, being found in significantly lower values in the remaining fields. The contents of S were significantly higher in field 2 but did not vary among the remaining experimental fields. The amount of K did not change significantly in fields 3 and 4 but remained lower than in fields 1 and 2 (between 10.45% and 45.11%, relatively to fields 1 and 2). Calcium showed significantly higher values in field 2 (ca. 5.7 fold), whereas field 3 had the lowest value. The levels of Fe did not vary significantly in fields 2 and 4 but remained lower relative to the other fields. The amount of Mn was significantly different among the experimental fields according to the following pattern: field 3 > field 1 > field 4 > field 2. Moreover, although Zn content in the soils of the four wheat fields were Zn-sufficient for keeping an adequate plant status [37], it varied significantly and followed the pattern: field 1 > field 3 > field 2 > field 4. Contaminants, like As and Cd, were also found in considerable quantities on the experimental fields (except field 4, for As). Contents of Mg and P remained lower than 1500 and 200 mg/kg, respectively.

### 2.2. Zinc Contents and Deposition in Grain Tissues

**Figure 1:**
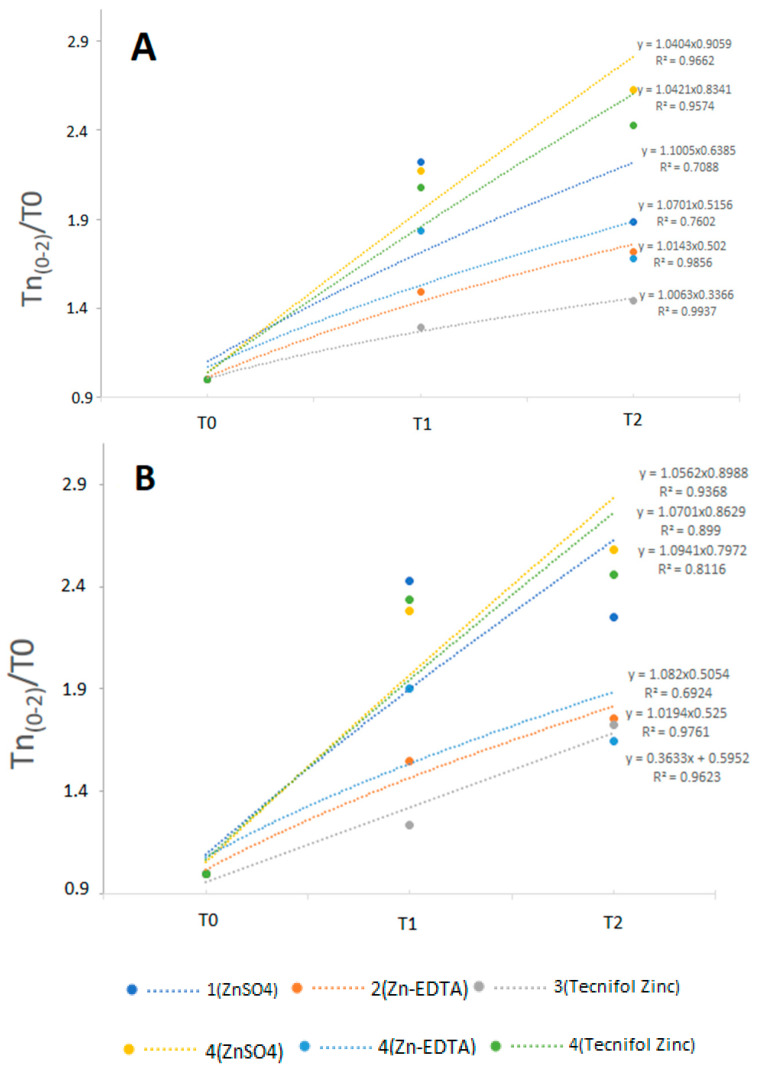
Ratio between the average value of Zn concentration in each treatment and the control (T0), as well as the relative trend line for all the wheat fields of Roxo (**A**) and Paiva varieties (**B**). Average values of Zn concentrations and trend lines of each wheat field are expressed in circles and lines.

In each experimental field, Zn contents in the whole wheat grains showed higher values in Roxo (Table 2), with the highest values being found in T2 (except for fields 1 and 4—Zn-EDTA with T1 showing the highest value, although only significant in field 1). In all treatments, Paiva further systematically showed the lowest Zn content in T0. In field 4, the highest Zn content was found after application of ZnSO_4_ in T2 (Roxo) and the lowest in T0 of Paiva. Among the wheat fields, T0 of Roxo only showed a significant higher Zn content in field 3, whereas T1 prevailed in fields 1 and 4, followed by fields 3 and 2, and T2 showed the highest content in field 4 (ZnSO_4_), followed by fields 4 (Tecnifol Zinc) and 1 and fields 2 and 4 (Zn-EDTA). Relative to T0 of Paiva, field 3 showed the highest Zn contents, followed by fields 4 (ZnSO_4_), 2 and 4 (Zn-EDTA) and therefore fields 4 (Tecnifol Zinc) and 1, whereas T1 prevails in fields 1 and 4, followed for 3 and 2, and in T2 the highest values were found in field 4 (ZnSO_4_), followed by field 3 and fields 4 (Tecnifol Zinc), 2 and finally field 1. Moreover, it was interesting to notice that in all fields Zn contents in Roxo and Paiva grains (Figure 1A,B) showed trendlines following a similar order of decreasing accumulation kinetics: 4 (ZnSO_4_) > 4 (Tecnifol Zinc) > 4 (Zn-EDTA) > 1 (ZnSO_4_) > 2 (Zn-EDTA) > 3 (Tecnifol Zinc). Besides, under similar soil characteristics (i.e., field 4), for both varieties, the highest kinetics for Zn accumulation occurred with ZnSO_4_, whereas Tecnifol Zinc and Zn-EDTA reversed their position relative to fields 2 and 3.

**Figure 2:**
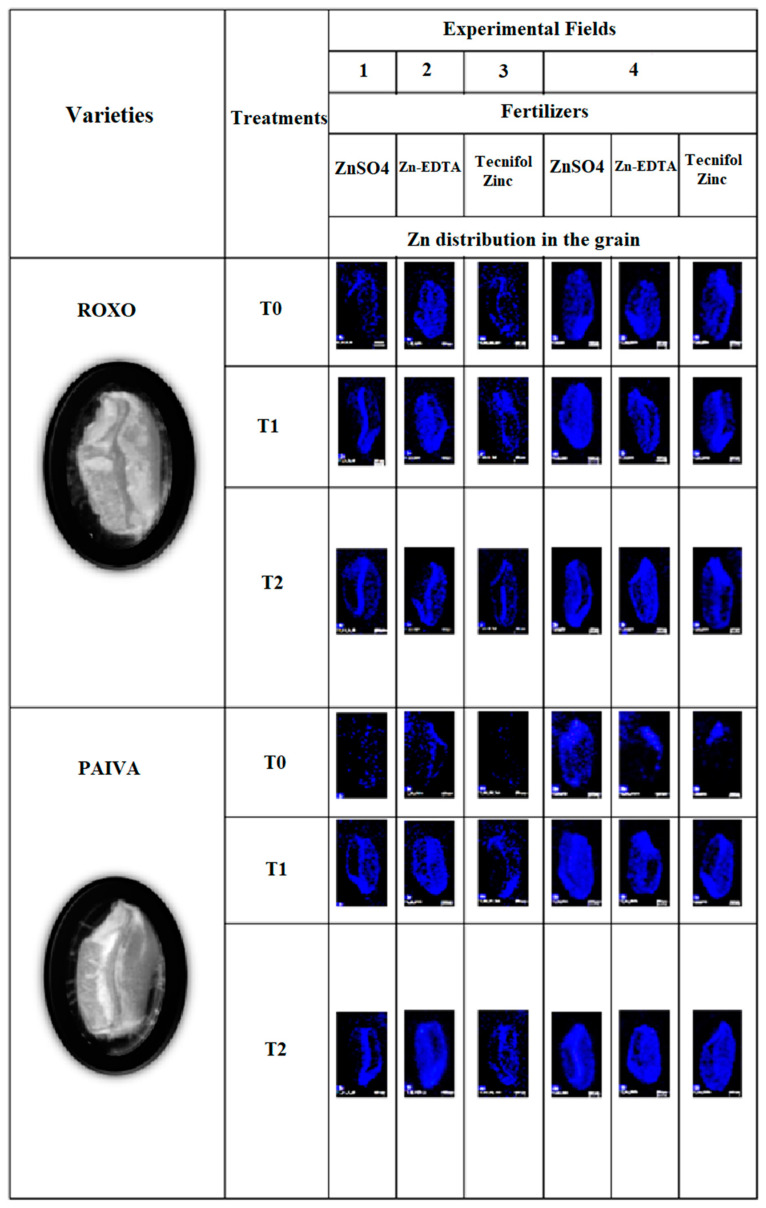
Elemental maps of *Triticum aestivum* L. grains, of Roxo and Paiva varieties, from the experimental fields 1–4, with the spatial distribution of Zn (in blue) in layered images. For the same fertilizer (ZnSO_4_, Zn-EDTA and Tecnifol Zinc): T0 = control; T1, T2 correspond, respectively, to initial and upper concentration.

At harvest, Zn contents on the mature grain of both varieties in general increased as the application of Zn fertilizer had risen, being preferably located in the embryo and vascular bundle and less accumulated in aleurone (Figure 2).

### 2.3. Grain Yield, Test Weight, Thousand Kernel Weight, Moisture and Macroscopic Aspects

Independent of each treatment, relative to Roxo, in fields 1 and 3 significantly higher grain yields were found in Paiva, after application of ZnSO_4_ and Tecnifol Zinc, respectively (Table 3). Moreover, in field 2, using Zn-EDTA as a fertilizer, significant differences could not be found among treatments for both varieties. In field 4, among treatments, after application of ZnSO_4_ and Tecnifol Zinc, Paiva showed significantly higher grain yields (except T2 and T1 with ZnSO_4_ and Tecnifol Zinc, respectively). In each variety and experimental field, significant differences were not found among treatments.

Test weight only showed significantly lower values in T2 of Roxo (field 1), T1 of Paiva (only relatively to all treatments in Roxo of field 3) and T2 of Roxo (field 4—Zn-EDTA), whereas Paiva revealed significantly higher values relative to T2 of Roxo (field 4—Zn-EDTA) (Table 3). In each variety and experimental field, significant differences could not be found.

In field 1, moisture content was significantly lower for T1 of Roxo (relatively to T1 and T2 of Paiva), whereas in field 3 significantly lower and higher values were found for T2 of Roxo (only relatively to T1 of Paiva) and T1 of Paiva (only relatively to all treatments of Roxo), respectively (Table 3). Significant variations could not be found in field 2, for both varieties and treatments. In field 4, after application of ZnSO_4_, significantly lower values were obtained for T1 of Roxo (relatively to T0 of Paiva), whereas after application of Zn-EDTA and Tecnifol Zinc, differences could not be found in the related treatments for both varieties.

In field 1, Thousand Kernel Weight (TKW) was significantly higher in all treatments of Paiva (although not for T0), whereas in field 2 the value for T2 remained lower for T2 of Roxo (but only significantly relatively to T0 e T1 of Paiva) (Table 3). In field 3, all treatments of Paiva remained significantly higher (except relatively to T1 of Roxo). In field 4, with ZnSO_4_, T0 and T1 of Roxo and Paiva respectively showed significantly higher values for TKW, relative to the other treatments (except T0 in Paiva), whereas after application of Zn-EDTA or Tecnifol Zinc, all treatments of Paiva showed higher values relative to Roxo.

Considering all experimental fields, it was found that the grain yield of Roxo in field 4, after application of ZnSO_4_, showed the highest value for T0 (although only significant relative to T0 of fields 2 and 3, after application of Zn-EDTA and Tecnifol Zinc, respectively), but T1 and T2 did not vary significantly (Table 3). Relative to Paiva, the grain yield of T0 revealed the highest values in fields 1 and 4, with application of ZnSO_4_ (although only significant relatively to field 2 and 4, with application of Zn-EDTA), whereas field 2 revealed the significantly lower value after application of Zn-EDTA, and T2 showed the lowest value in field 2, followed by field 4 (both treated with Zn-EDTA, being significantly different).

In field 1, test weight only varied significantly between the control of Paiva (revealing minimum values) and T2 of Roxo, whereas field 2 and 4 (with ZnSO_4_ spraying) did not vary significantly, and field 3 showed minimum values in Paiva (without significant differences among treatments) (Table 3). In field 4, after spraying with Zn-EDTA and Tecnifol Zinc, minimum and maximum values were found for T2 of Roxo and T0 of Paiva, respectively. In all the wheat fields, T0, T1, T2 of Roxo only revealed significantly lower values in field 3, whereas all these treatments of Paiva showed significantly lower values in fields 2 and 3.

In field 1, relative to T1 and T2 of Paiva, moisture showed significantly lower values in T1 of Roxo, whereas significant differences were not found in field 2 (Table 3). In field 3, relative to T0 and T1, significantly lower values occurred in T2 of Roxo, while for T0 and T2 of field 4, minimum and maximum values were found in Roxo (Zn-EDTA) and Paiva (Tecnifol Zinc), respectively. In field 4, relative to fields 1, 2 and 3, independent of the applied fertilizer, Roxo showed significantly higher moisture for T0 and T2, whereas T1 did not vary significantly among the experimental fields. T0 (Control) of Paiva showed, relative to fields 1, 2 and 3, significantly higher moisture in field 4 (independently of the applied fertilizer), with field 2 displaying minimum values. A similar pattern occurred with T1, yet T2 revealed significantly lower values in field 2, followed by field 1 and 3, whereas field 4 showed maximum values after application of Tecnifol Zinc.

Field 1 revealed minimum values of TKW for T1 and T2 of Roxo, whereas field 2 showed lower values for T2 of Paiva and, in field 3, higher values were found in all treatments of Paiva (Table 3). In field 4, T0 and T1 of Roxo and Paiva sprayed with ZnSO_4_ and T0, T1 and T2 of Paiva pulverized with Zn-EDTA and Tecnifol Zinc, did not vary significantly. All the remaining treatments of both varieties, submitted to the different fertilizers, did not vary significantly among them. Comparing the TKW of each treatment, in Roxo, among the different experimental fields, it was found that the lowest values of T0 occurred in field 4 (sprayed with Zn-EDTA and Tecnifol Zinc), whereas the highest were found in fields 1, 2 and 4 (pulverized with ZnSO_4_). Moreover, in Roxo, T1 kept the highest values in fields 2 and 4 (sprayed with ZnSO_4_), followed by fields 1 and 4 (pulverized with Zn-EDTA) and field 3. T2, of Roxo, revealed the highest values in field 4 (pulverized with ZnSO_4_), followed by field 1, 2 and 4 (sprayed with Zn- EDTA and Tecnifol Zinc). Concerning Paiva, the highest values of TKW were found in field 1 and 4 (fertilized with Zn-EDTA and Tecnifol Zinc), whereas the lowest occurred in field 3. In Paiva, T1 showed the highest values in fields 1, 2 and 4 (with all fertilizers), whereas T2 revealed the highest values in fields 1 and 4 (sprayed with Tecnifol Zinc) and the lowest in field 3.

**Figure 3:**
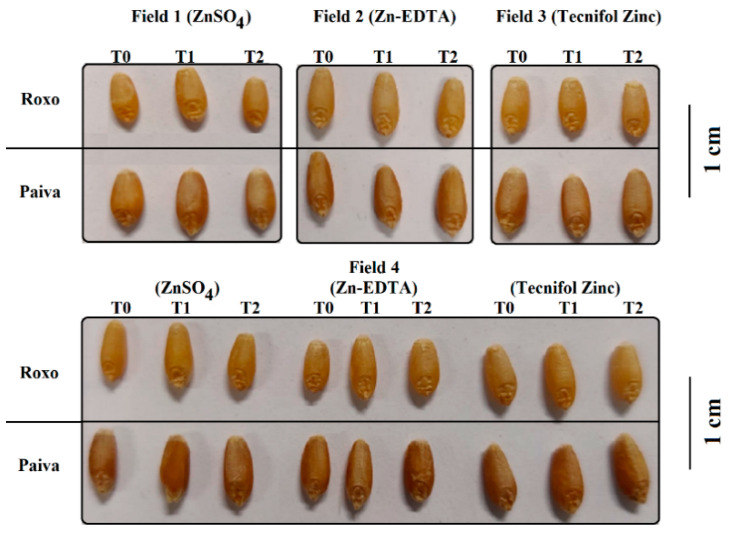
Macroscopic aspects of *Triticum aestivum* L. grains, of each treatment of Roxo and Paiva varieties, from the experimental fields 1–4. For the same fertilizer (ZnSO_4_, Zn-EDTA and Tecnifol Zinc): T0 = control; T1, T2 correspond, respectively, to initial and upper concentration.

At a macroscopic level, visual symptoms of grains deficiency or toxicity (i.e., deformed or shrunken) could not be found among treatments in each experimental field or genotype (Figure 3).

### 2.4. Ash Contents and Colorimetric Parameters of Whole Wheat Flour

In field 2, ash content of whole wheat flour did not vary significantly among treatments of both varieties and, in field 3, only T0 of Roxo varied significantly (Table 4). In field 1, ash showed significantly higher and lower values in T0 and T2 of Paiva, whereas in field 4 Roxo presented for T0, T1 and T2 significantly higher values and T1 and T2 of Paiva at the lowest levels. Among all T0 of Roxo, in all the experimental fields, the trend of ash content was fields 3 and 4 (ZnSO_4_) > field 1 > field 4 (Zn-EDTA and Tecnifol Zinc) > field 2, whereas for T1 were fields 1, 3 and 4 (ZnSO_4_) > field 4 (Zn-EDTA and Tecnifol Zinc) > field 2 and for T2 were fields 4 (ZnSO_4_) and 1 > field 3 and 4 (Zn-EDTA and Tecnifol Zinc) > field 2. Among all T0 of Paiva, in all the experimental fields, the trend of the ash content was field 1 > fields 2, 3 and 4 (ZnSO_4_ and Zn-EDTA) > field 4 (Tecnifol Zinc), whereas in T1 it was fields 3 and 4 (ZnSO_4_ and Zn-EDTA) > field 1 > fields 2 and 4 (Tecnifol Zinc) and for T2 was fields 3 and 4 (ZnSO_4_) > fields 1, 2 and 4 (Zn-EDTA and Tecnifol Zinc).

**Figure 4:**
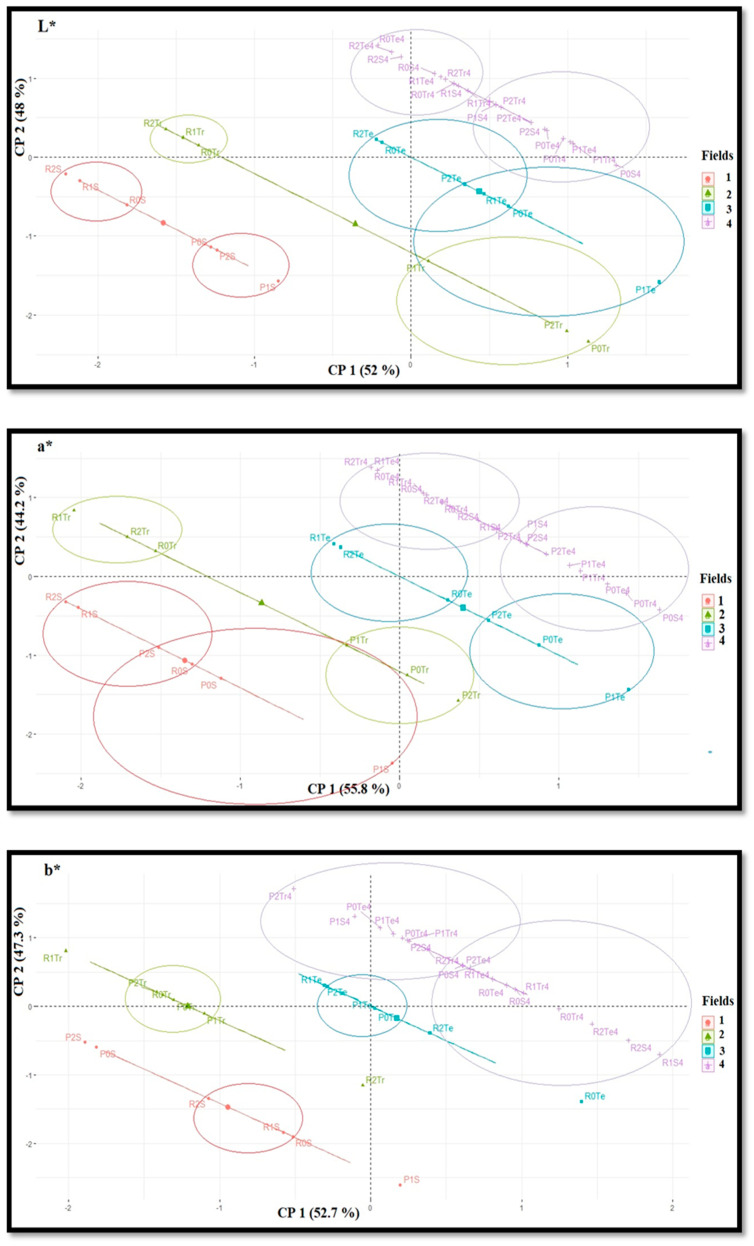
Attributes distribution formed by first and second principal components (CP 1 and CP 2, respectively) of colorimeter parameters L*, a* and b*—CIELab System (*n* = 12) of whole wheat flour, of fields 1, 2, 3 and 4 of *Triticum aestivum* L., varieties Roxo (R) and Paiva (P). Concentrations of treatments from the same fertilizer are divided into: control (0); C1 (1), C2 (2) corresponding, respectively, to an initial application and the upper application of the fertilizers ZnSO_4_ (S), Zn-EDTA (Tr) and Tecnifol Zinc (Te) (statistical analysis by software R version 3.6.3).

Color parameters of whole wheat flour, among treatments, of Roxo and Paiva, from the different experimental fields, displayed significant variations (Table 4). In field 1, T0 and T2 of Paiva revealed the highest and the lowest values for L* (i.e., lightness), respectively. In field 2, L* did not show significant variations for Zn-EDTA-treated Roxo and Paiva, whereas field 3 only revealed significantly higher values for T0 of Roxo. Field 4 revealed the highest values of L* in Roxo treated with ZnSO_4_, whereas the lowest were detected with Tecnifol Zinc in both varieties and in Paiva sprayed with Zn-EDTA. Among all T0 of Roxo, in all the experimental fields, the trend of L* was fields 1, 2 and 4 (ZnSO_4_ and Tecnifol Zinc) > fields 3 and 4 (Zn-EDTA), whereas for T1 and T2 it was fields 1, 2, and 4 > field 3. Relative to Paiva, the trend of L* for T0 was fields 1, 3 and 4 > field 2, whereas for T1 it was fields 1 and 4 > field 2 > field 3, and for T2 was fields 1, 3 and 4 > field 2. In field 1, a* (i.e., red–green transitions) revealed significant differences, for T1 of Paiva and T2 of Roxo showing the highest and lowest values, respectively. Fields 2 and 3 showed, relative to Roxo, the highest a* values in all treatments of Paiva, whereas field 4 showed the significantly higher values for T0 sprayed with ZnSO_4_ and Zn-EDTA and the lowest for T0 of Roxo (ZnSO_4_) and for all treatments of Roxo (Zn-EDTA and Tecnifol Zinc). Among all T0 of Roxo, in all the experimental fields, the trend of a* was fields 1 and 3 > field 4 > field 2, whereas for T1 and T2 was fields 3 and 4 > field 1 > field 2. Among all T0 of Paiva, in all the experimental fields, the trend of a* was fields 2, 3 and 4 > field 1, whereas for T1 it was fields 1, 2, 3, 4 (Zn-EDTA) > field 4 (ZnSO_4_ and Tecnifol Zinc), and for T2 it was fields 2, 3 and 4 > field 1. In field 1, b* (i.e., yellow–blue transitions) revealed significant differences, for T0 and T1 of Paiva showing the lowest and highest values, respectively, whereas in field 2 significant differences could not be found. In field 3, for b*, only T0 of Roxo showed significantly higher values, whereas in field 4 the highest and the lowest significant values were found for T1 (ZnSO_4_) of Roxo and T2 (Zn-EDTA) of Paiva. Among all T0 of Roxo, in all the experimental fields, the trend of b* was fields 1, 3 and 4 (Zn-EDTA and Tecnifol Zinc) > fields 2 and 4 (ZnSO_4_), whereas for T1 it was fields 1 and 4 > field 3 > field 2, and for T2 it was fields 1, 3 and 4 (ZnSO_4_ and Tecnifol Zinc) > fields 2 and 4 (Zn-EDTA). Among all T0 of Paiva, in all the experimental fields, b* did not vary significantly, whereas the trend of b* for T1 it was fields 1 and 3 > fields 2 and 4, and for T2 it was fields 1, 3 and 4 (ZnSO_4_ and Tecnifol Zinc) > fields 2 and 4 (Zn-EDTA). Through an analysis of cluster definition of color parameters, it was found that in each experimental field specific clusters were defined for parameters L*, a* and b* (Figure 4). For parameter L* and a*, all wheat fields showed an individual cluster for each genotype. Moreover, parameter b* showed individual clusters only in field 4, whereas in fields 2 and 3 these were identified only for Paiva and in field 1 for Roxo.

## 3. Discussion

### 3.1. Soil Aptitude for Zn Biofortification

Crop biofortification must directly satisfy plants needs to produce healthy edible portions. Yet, the nutrient density of seed is dependent [38] on inherent fertility status, soil properties (namely organic, bioavailability of macro and micronutrients and moisture), crops species and cultivars (i.e., different genotypes can differ in phonological behavior and interaction due to genetic variation). Accordingly, four bread wheat fields were selected, to assess the interactions between nutrients contents, pH, electrochemical conductivity and organic matter in soils, during Zn enrichment, taking into account that, depending on soil properties, wheat crop is estimated to remove a broad range of Zn (about 66–209 g of Zn for every 2 tons of wheat grains) [39].

Considering that Zn deficiencies (i.e., Zn content of unpolluted soils ranges between 10–80 mg/kg) prevail in about 50% of soil samples collected worldwide [40,41], the four experimental fields were further chosen to assess the implications of heterogeneous Zn contents in soils on the wheat grain biofortification index, which revealed notorious deviations (Table 2). Indeed, the kinetics of Zn uptake by roots was conditioned by significantly different contents of this nutrient in the soils of the four wheat fields (Table 1), which decreased in the following order: field 1 > field 3 > field 2 > field 4. Nevertheless, the bioavailability of Zn in soils is controlled by both absorption–desorption reactions and solubility relations, and the soil solution and solid phase are mainly involved in the absorption–desorption and dissolution precipitation reactions of Zn in soils [42]. Accordingly, low levels of pH (i.e., below 7) and organic matter in the soils augments Zn solubility and availability (as higher organic compounds in soils determines the synthesis of organic complexes), with low electrochemical conductivity, leading to shorter expenditure for water uptake by roots [21,43,44,45]. Therefore, considering these parameters, Zn uptake by roots in the wheat fields become favored according to the following trend: field 4 > field 3 > field 1 = field 2 (Table 1). In addition, Ca and S display an antagonist effect on Zn solubility, since this nutrient uptake follows across the plasma, which covers root cells as Zn^2+^ [46], while it is also permeable to plasma membrane Ca^2+^ channels [47]. Moreover, the antagonistic interaction between Zn and Fe, as well as with Mn, favors the related oxides synthesis, triggering higher availability for root uptake [48]. Therefore, these soil interactions suggest a hold-up Zn availability in the wheat fields, according to the following trend: field 3 > field 1 > field 4 > field 2 (Table 1). Moreover, although K contents showed the lowest values in fields 3 and 4, and Cu only showed significantly lower values in field 1 (Table 1), as their dynamics in soils depends on the magnitude of equilibrium among various chemical forms, a direct interaction on Zn availability is uncertain. A similar perspective applies to Cd and As, since the interactions with Zn for uptake and translocation in plants appears to be somewhat controversial [48,49,50], although Cd uptake might decrease with increasing Zn fertilization [51].

Considering all the studied soil determinants (Table 1), our data indicated that wheat field 3 showed the better edaphic conditions (although with the highest levels of organic matter, pH below 7, high Zn, Fe and Mn contents and low values of Ca and S). Thereafter, better conditions were found in field 1 (showing the highest contents of Zn, high levels of Fe and Mn and the significantly lower amounts of organic matter, Ca and S, but with pH higher than 7). Field 4 had worse edaphic conditions (in spite of pH below 7 and with the lowest amount of organic matter, with sharp limitations of Zn, Mn and Fe), followed by field 2 (revealing high contents of organic matter, low amounts of Zn, Fe and Mn and the highest levels of Ca, S and pH (Table 1)).

### 3.2. Ash, Zinc Contents and Deposition in Grain Tissues

Ash contents, as an indication of total nutrient concentration, did not reveal a positive correlation with Zn accumulation (Table 2 and Table 4), which can mostly be attributed (in spite of the well-known relations with Fe and Cu uptake [52,53]) to heterogeneous interactions among macronutrients deposition in the grains of both genotypes. Thus, our data point out that nutrient deposition is closely linked with the heterogeneous characteristics of genotypes Roxo and Paiva (which determine different kinetics of root uptake) and specific characteristics of soils composition in the four bread wheat fields (Table 1). Indeed, it has long been known that Zn interacts positively, namely with K, and negatively with P and Ca [52,53]. Additionally, top dressing all the wheat fields with N also positively interacted with Zn magnification in the wheat grains by improving the grain protein amounts and thereby escalating the sink strength in grains for Zn [54]. Nevertheless, as Zn uptake is mostly controlled by its transport across the plasma membrane, which is largely metabolic-dependent and genetically controlled, Zn-efficient genotypes may be able to maintain structural and functional stability of their root-cell plasma membranes better than Zn-inefficient genotypes under Zn deficiency [55]. In this context, Zn accumulation in Roxo grains, which is an old variety, remained consistently higher, relative to the levels in the Paiva grains (Table 2), which agrees with [56]. Indeed, despite the breeding advances to increase grain yields, newly bred varieties are showing a limited capacity to enhance nutrient uptake efficiency [56].

Nutrient density per unit of grain dry weight is highly important for estimating grain quality [57]. Therefore, at critical growth stages of a crop, proper supply of micronutrients improves grain quality and the health status of human consumers [35]. Without Zn pulverization, this nutrient accumulation in the grain is only linked to its supply during developing, either by direct uptake from the soil through membrane bound transporters [58] followed by xylem loading and unloading and vacuolar sequestering and remobilization [59], or by remobilization from leaves. Accordingly, soil properties of the four wheat fields determined grain accumulation of T0 in both varieties only through Zn roots uptake kinetics (Table 1 and Table 2). Additionally, due to the top dressing of all fields with N, root uptake and transport of Zn, via chelation with nitrogenous compounds, improved proportionately Zn deposition in the grains, with N-levels [27]. In this context, as the control treatments (T0) of Roxo and Paiva genotypes were not sprayed with the Zn fertilizers, it was interesting to note that the highest accumulation of this nutrient in the grain correlated positively with soil properties of field 3 (Table 1 and Table 2). Roxo grains of field 1 further showed the second highest Zn content, which therefore also interacted with soil properties. Additionally, field 4 and 2 showed similar values of Zn in the grain and again a positive relation was found. Facing Zn accumulation of Paiva grains in T0, field 1 revealed the lowest value, which suggested the prevalence of phenotype specificity, namely linking a decreased ratio between Zn uptake and higher grain yield (Table 3).

Zinc fertilization, which has high phloem mobility in wheat [60], is well-known to increase this nutrient accumulation in the grain and, consequently, the whole flour zinc concentration in wheat, either by soil or foliar application, or by combining soil and foliar zinc applications [28,61,62]. Yet, considering that the timing of a micronutrient foliar application delineates its effectiveness to increase grain contents, in our experiment, spraying with Zn-EDTA and Tecnifol Zinc occurred at booting, heading and grain milk stages, whereas pulverization with ZnSO_4_ took place only at booting and heading to avoid toxicity symptoms. Foliar Zn application, which is much effective than this nutrient application in the soil, triggered the highest kinetics of Zn accumulation in the grains of Roxo and Paiva under similar edaphic conditions (i.e., field 4). Accordingly, our data pointed that ZnSO_4_ is the best spraying fertilizer for wheat biofortification, whereas Zn-EDTA is less effective (Figure 1). Therefore, our results do not support the report of [63], stating that the effective period for biofortification operation with Zn is the milking stage to the grain filling stage. However, with the report of [62], we found that, relative to ZnSO_4_, foliar spraying with Zn-EDTA resulted in lower values of zinc in grains (32.3 mg/kg—ZnSO_4_ and 29.0 mg/kg—Zn-EDTA), when 0.5 kg Zn/ha of ZnSO_4_ and 0.1 kg Zn/ha of Zn-EDTA were applied three times during the grain filling stage. Eventually, foliar spraying with Zn-EDTA is less effective than ZnSO_4_ because fertilizers have a distinct leaf penetration capacity, as Zn-EDTA has a carbon skeleton with higher dimensions [62]. Similarly [61], after spraying with 1.3, 2.2 and 3.0 kg/h of ZnSO_4_·7H_2_O during the wheat life cycle, we also found an enhancement of Zn concentration in the grains, which as a Zn foliar application is phloem-mobile, and might be a high capacity of translocation into wheat grains. Additionally, considering that the soil of field 4 had the lowest content of Zn, being further limited by low organic matter and nutrient interactions (Table 1), our data indicated that the efficiency of Zn biofortification is largely determined by foliar fertilization (Table 1; Figure 1). Indeed, despite the soil characteristics of field 3, after spraying with Tecnifol Zinc, both genotypes had the lowest accumulation kinetics in the grains (Figure 1). Nevertheless, the highest efficiency of Zn spraying must also be linked to soils with high amounts of this nutrient to achieve the best biofortification index (Table 1 and Table 2; Figure 1).

At harvest, Zn contents on the mature grain of both varieties was preferably located in the embryo and vascular bundle and less accumulated in aleurone (Figure 2), a pattern also reported by [64,65]. Thus, our data indicated that, for both genotypes, whole wheat flour enriched with Zn becomes a better option for human consumption because after milling Zn-rich parts (i.e., the aleurone and embryo) are mostly removed and only the endosperm remains, making refined wheat flour poorly Zn-enriched.

### 3.3. Grain Yield, Test Weight, Thousand Kernel Weight (TKW) and Colorimetric Parameters

Despite the significantly different Zn contents in the soils of the four experimental fields, biofortified wheat grains did not reveal typical symptoms [66] of yield reduction (Table 3; Figure 3). Additionally, in all wheat fields, and therefore independent of the foliar fertilizer applied (Table 3), grain yield of each genotype did not show significant differences among treatments (except the control of Roxo in field 4), with Roxo showing a similar tendency to that reported by [28] after foliar spraying with ZnSO_4_·7H_2_O. We found in fields 2 and 4, as also [62], through pulverization with Zn-EDTA, further working with bread wheat, did not find significant differences of grain yield/test weight. Yet, [61,67] it was found in an opposite trend for grain yield and TKW, which could be attributed to different growth conditions and genotype specificity. Nonetheless, following [68], in general, in both genotype top-dressings, all wheat fields with N boost both grain yield and protein amounts. Moreover, dry matter accumulation and yield attributing characters mostly differ when different cultivars were selected based on their genotypic sequencing. Indeed, independent of each treatment, the consistently higher values of grain yield of Paiva, relatively to Roxo, found in fields 1, 3 and 4 (except T2 and T1 with ZnSO_4_ and Tecnifol Zinc, respectively), through pulverization with ZnSO_4_ and Tecnifol Zinc, was the result of wheat breeding (i.e., Roxo and Paiva are old and modern varieties, respectively). Paiva was developed for achieving higher grain weight and TKW. Nevertheless, independent of the edaphic characteristics of the experimental fields, and although in most treatments significant differences could not be found, the highest grain yield was obtained through pulverization of Roxo and Paiva with ZnSO_4_, whereas foliar spraying with Zn-EDTA consistently gave the lowest values (Table 3). Besides, to some extent, similar trends were also found for test weight moisture content and TKW.

To assess agricultural crop yield, relative to the environmental and technological factors such as fertilizer dosage, imagery techniques can be used. Following the system of the Comission Internationale d’Éclaire (CIE), a correlation between Zn enrichment and the color parameters L*, a* or b* could not be found (Table 4), as in spite of the variations detected in fields 1, 2 and 3, these were not confirmed in field 4. Moreover, in each experimental field, individual clusters were found (Figure 4), which indicated that the conjunction of edaphic characteristics and types of foliar fertilizers can define specific patterns of L* and a* parameters for both genotypes.

## 4. Materials and Methods

### 4.1. Experimental Fields

Two Portuguese varieties of *Triticum aestivum* L. (Roxo and Paiva) obtained in the breeding Programme of the National Institute for Agriculture and Veterinary Research (INIAV), located in Elvas, Portugal, were cultivated in four experimental fields. Wheat fields were located at 37°57′09.68″ N; 7°30′26.82″ W (field 1); 37°57′27.59″ N; 8°08′05.50″ W (field 2); 37°58′56.10″ N; 7°44′18.38″ W (field 3) and at 38°01′52.38″ N; 7°52′53.72″ W (field 4).

Fields were sown at 30 December of 2018, with a rate of 350 seeds/m^2^, and the harvest of fields 1 and 3 took place at 26 June of 2019, while for fields 2 and 4 this period finished on 12 July and 27 June of 2019, respectively. During plant cycles, the average maximum and minimum temperatures were 22 °C and 11 °C, respectively (with maximum and minimum temperatures of 39 °C and 0 °C, respectively). During this period, the maximum and minimum values of air humidity were 100% and 0%, respectively (with maximum and minimum averages ranging between 69% and 11%, respectively). The total rainfall accumulation was about 5.43 mm (with a daily maximum of 1.85 mm).

Before sowing, fields were fertilized with 50 kg Zn/ha. NPK fertilization was applied before sowing (Foskamónio 12–24–22, 250 kg/ha, with 2.5% N-nitric and 9.5% N-ammoniacal, 20–24% of phosphorus pentoxide soluble in citrate of neutral ammonium and water, 12% water soluble potassium oxide) and N was additionally applied in two top dressing (Nergetic DS 24/5–14, 200 kg/ha, with 24% total nitrogen, 12% nitrous nitrogen, 12% ammoniacal nitrogen, 5% water soluble CaO, 14% total SO_3_ and 0.03 water soluble B). Fields were sown in a randomized block design with four repetitions, where fields 1, 2 and 3 presented 24 plots, with an area of 12 m^2^ (10 m × 1.2 m) each, comprising 0.4 m between plots and 3 m between repetitions. Field 4 comprised 72 plots (each 24 plots for a different zinc fertilizer application) with an area of 9.6 m^2^ (8 m × 1.2 m), with 0.4 m rows between plots and 2 m between repetitions. Each of the four experimental fields were divided into two sections and cultivated with both bread wheat varieties (Roxo and Paiva). During April and May, the agronomic biofortification comprised Zn foliar pulverization at booting, heading and grain milk stages (except on the plots sprayed with ZnSO_4_, as in this case foliar applications took place only at booting and heading). Each fertilizer was applied with three different concentrations. In field 1, ZnSO_4_ was applied (two sprays—each with 0-control, 8,1 and 18.2 kg/ha; total applied—0 for the control and 16.20 and 36.40 kg/ha); field 2 was sprayed with Zinc-EDTA (three sprays, each with 0-control, 2.1 and 4.2 kg/ha; total applied—0 for the control and, 6.3 and 12.60 kg/ha); field 3 was pulverized with Tecnifol Zinc (three sprays, each with 0-control, 1.3 and 2.6; total applied—0 for the control, 3.9 and 7.8 kg/ha); field 4 was sprayed with all the three fertilizers (ZnSO_4_, Zinc-EDTA and Tecnifol Zinc, with the concentrations mentioned above).

### 4.2. Soil Analysis

In fields 1, 2 and 3, soil samples (9) were collected from surface to a 30 cm deep, using a rectangular grid of 23 × 22 m. In field 4, soil samples were collected from surface to a 30 cm deep, using a rectangular grid of 38 × 31 m. Approximately 100 g of each sample was sieved, using a 2.0 mm mesh, to remove coarse materials, stones and other debris. Sample weight was recorded and, after drying for 24 h at 105 °C, samples were taken to desiccation for 1 h, followed by determination of dry mass and moisture. Thereafter, samples were placed in a muffle, heated for 4 h, at 550 °C (until constant weight), and removed after cooling until 100 °C. Samples were then placed in a desiccator (for about 1 h, until room temperature) and weighted to assess the percentage of organic matter.

Soil electrical conductivity and pH were determined using potentiometer, and after sample mixing (at a ratio of 1:2.5 g_soil_ mL^−1^_water milli-q_, for 1 h), keeping thereafter the mixture at 25 °C, for 30 min in a thermal bath, followed by decantation of the supernatant [69].

Mineral content in soils were determined using an XRF analyzer (model XL3t 950 He GOLDD +), under helium atmosphere [70].

### 4.3. Zinc Contents and Deposition in Grain Tissues

Zinc content in the whole wheat flour was determined using an XRF analyzer (model XL3t 950 He GOLDD +) under helium atmosphere [70]. For each sample, measurements were carried out in triplicate with emission of radiation for 180 s. For data analysis, the software NITON Data Transfer (XL 3t-36653) was used.

A micro-Energy Dispersive X-Ray Fluorescence system (μ-EDXRF) (M4 Tornado™, Bruker, Germany) was used to assess Zn deposition in grain tissues collected at harvest [65]. The X-ray generator was operated at 50 kV and 100 μA without the use of filters, to enhance the ionization of low-Z elements. To a better quantification of the element, a set of filters between the X-ray tube and the sample, composed of three foils of Al/Ti/Cu (with a thickness of 100/50/25 μm, respectively) was used. All the measurements with filters were performed with a 600 μA current. Detection of fluorescence radiation was performed by an energy-dispersive silicon drift detector, XFlash™, with 30 mm^2^ sensitive area and energy resolution of 142 eV for Mn K_α_. To better measure the distribution mapping of Zn, grains were longitudinally cut in half, along the crease tissue, with a stainless-steel surgical blade. Measurements were carried out under 20 mbar vacuum conditions and performed directly on one side of the grains. These point spectra were acquired for 200 s.

### 4.4. Grain Yield, Test Weight, Thousand Kernel Weight, Moisture and Macroscopic Aspects

After harvest, the grain was threshed and weighted. Grain yield was expressed as kg of dry matter per ha [12]. Additionally, test weight (expressed as kg/hL) was analyzed in a cytometer, and thousand kernel weight (TKW) was further determined.

Determination of grain moisture content was based on [71]. The Infratec™ 1241 Grain Analyzer (Foss, Denmark) was used and 800 g of grain was added.

Macroscopic aspects were obtained using a 48 OIS + 8+5 + 2 MP Quad Camera, coupled with a 32 + 8 MP Dual Front Camera.

### 4.5. Ash and Colorimetric Parameters of Whole Wheat Flour

Ash content in wheat flour was determined according to [72]. Each sample (5 g), in triplicate, was weighted and incinerated for 2 h, at 900 °C, followed by desiccation until room temperature. Samples were therefore weighted, and the ash content was determined.

The colorimetric parameters (chromaticity parameters a* and b* and lightness, L*) of the whole wheat flour were analyzed (in triplicate) using a Minolta CR 400 colorimeter (Minolta Corp., Ramsey, NJ, USA), coupled to a sample vessel (CR-A504) [73]. The system of the *Comission Internationale d’Éclaire* (CIE) was applied using the illuminate D_65_. Parameter a* indicated color variations between red (+60) and green (−60), while the parameter b* color varied between yellow (+60) and blue (−60). The parameter L* represented the lightness of each sample, translating the variation of the tonality between dark (0) and light (100). The approximation of these coordinates to the null value translated neutral colors like white, grey and black.

### 4.6. Statistical Analyses

Data were statistically analyzed using software R (R version 3.6.3). Statistical analysis included a principal component analysis and One-Way and Two-Way ANOVA (*p* ≤ 0.05) to assess significant differences. Based on the results, a Tukey’s test for mean comparison was performed, considering a 95% confidence level. Data normality and homogeneity of variance was also carried out.

## 5. Conclusions

Without foliar spraying, the edaphic characteristics of the wheat fields determined grain enrichment in Zn through this nutrient solubility and availability in the soil, which in turn is conditioned by interactions among a pH lower than 7, organic matter (which can develop organic complexes with Zn) and low electrochemical conductivity (that decreases water requirements). Additionally, Zn uptake by roots antagonistically interacted with other nutrients, namely Ca and S, and synergistically with Fe and Mn. Nevertheless, genotype characteristics of Roxo and Paiva further corroborated with the efficiency of root uptake kinetics. Moreover, for Zn biofortification, foliar pulverization with ZnSO_4_ became the best foliar fertilizer, whereas Zn-EDTA was the least effective. However, independent of the fertilizer applied, Zn accumulation prevailed in the embryo and vascular bundle and, to a lesser extent, in aleurone. Under the edaphic parameters and applied foliar fertilizers, in each wheat field and genotype, the grain yield and test weight in general were not negatively affected. Nonetheless, the different genotype characteristics of both wheat varieties triggered different yields when ZnSO_4_ (that gave the highest values in Paiva and Roxo) and Tecnifol Zinc were used. Screening Zn enrichment using, as referential, color parameters, could not be carried out, since a correlation between Zn enrichment in the grains and the color parameters L*, a* or b* did not occur. Moreover, lightness and red–green transitions were found to be a conjunction of genotype characteristics, fertilization types and edaphic conditions prevailing in each field.

## Figures and Tables

**Table 1:** Analysis of soils (0–30 cm) of fields 1–4 (*n* = 9 for electrochemical conductivity, pH, organic matter and moisture; *n* = 27 for quantification of chemical elements) of *Triticum aestivum* L., varieties Roxo and Paiva.

Experimental Field		1	2	3	4
**Electrochemical Conductivity**	**µS/cm**	507.44 ± 30.98 a	521.78 ± 36.69 a	368.56 ± 19.27 b	328.00 ± 18.84 b
**pH (H_2_0)**	**-**	7.67 ± 0.05 a	7.80 ± 0.03 a	6.85 ± 0.07 b	6.85 ± 0.07 b
**Organic Matter**	**%**	6.86 ± 0.06 a	7.07 ± 0.13 a	7.23 ± 0.42 a	5.32 ± 0.34 b
**Moisture**		22.47 ± 0.14 a	20.25 ± 0.65 ab	19.58 ± 0.35 b	15.09 ± 0.90 c
**S**		0.023 ± 0.001 b	0.025 ± 0.001 a	0.021 ± 0.001 b	0.020 ± 0.0004 b
**K**		0.622 ± 0.014 a	0.184 ± 0.009 b	0.065 ± 0.001 c	0.083 ± 0.0014 c
**Ca**		1.165 ± 0.060 b	6.331 ± 0.349 a	1.018 ± 0.027 b	1.103 ± 0.0284 b
**Mn**	**mg/kg**	755.58 ± 11.41 b	430.61 ± 21.81 d	840.74 ± 16.23 a	507.78 ± 25.69 c
**Fe**		38777 ± 376 b	21860 ± 936 c	46030 ± 419 a	22440 ± 746 c
**Cu**		46.91 ± 1.62 b	77.73 ± 7.21 a	87.04 ± 1.86 a	90.82 ± 3.00 a
**Zn**		59.81 ± 0.77 a	32.52 ± 2.95 c	40.94 ± 0.71 b	22.82 ± 0.61 d
**As**		10.98 ± 0.22 a	5.97 ± 0.41 b	5.61 ± 0.19 b	n.d.
**Cd**		24.18 ± 0.84 b	25.14 ± 0.87 b	36.37 ± 0.74 a	35.73 ± 1.43 a
**Mg**		<1500			
**P**		<200			

n.d. = Not detected. Different letters (a, b, c…) indicate significant differences, of each parameter among the different experimental fields (*p* ≤ 0.05).

**Table 2:** Average Zn contents ± SE (*n* = 12) of whole wheat flour of *Triticum aestivum* L., varieties Roxo and Paiva, from experimental fields 1–4, after fertilization ZnSO_4_, Zn-EDTA and Tecnifol Zinc.

Variety	Treatment	Experimental Field/Fertilizer					
		1	2	3	4		
		ZnSO_4_	Zn-EDTA	Tecnifol Zinc	ZnSO_4_	Zn-EDTA	Tecnifol Zinc
		Zn Contents (mg/kg)					
**Roxo**	**T0**	64.16 ± 2.516 ^d,B^	53.99 ± 1.270 ^d,B^	78.52 ± 3.298 ^cd,A^	59.99 ± 1.537 ^fghi,B^	54.16 ± 2.280 ^hi,B^	51.96 ± 5.479 ^hi,B^
	**T1**	142.5 ± 4.556 ^a,A^	80.60 ± 3.189 ^bc,C^	101.4 ± 1.277 ^b,AB^	130.2 ± 2.577 ^abc,AB^	99.57 ± 18.26 ^cde,AB^	107.9 ± 2.974 ^bcde,AB^
	**T2**	120.8 ± 3.386 ^b,BC^	92.79 ± 1.687 ^a,D^	113.1 ± 1.399 ^a,C^	157.8 ± 5.355 ^a,A^	90.95 ± 2.367 ^defg,D^	126.1 ± 0.785 ^abcd,B^
**Paiva**	**T0**	38.80 ± 1.138 ^e,D^	50.44 ± 2.018 ^d,B^	69.21 ± 3.446 ^d,A^	55.07 ± 1.372 ^ghi,B^	48.72 ± 1.892 ^hi,BC^	41.34 ± 1.499 ^i,CD^
	**T1**	94.11 ± 3.884 ^c,AB^	77.94 ± 2.659 ^c,B^	85.73 ± 1.003 ^c,B^	125.7 ± 7.887 ^abcd,A^	92.67 ± 17.67 ^def,AB^	96.65 ± 10.84 ^cde,AB^
	**T2**	87.39 ± 7.799 ^c,CD^	88.58 ± 0.842 ^ab,CD^	119.5 ± 1.810 ^a,B^	142.2 ± 3.011 ^ab,A^	80.28 ± 1.068 ^efgh,D^	101.6 ± 2.696 ^cde,C^

For the same fertilizer (ZnSO_4_, Zn-EDTA and Tecnifol Zinc): T0 = control; T1, T2 correspond, respectively, to initial and upper concentration. Letters a, b, c… indicate significant differences of Zn contents in the whole wheat flour among treatments within the same experimental field and letters A, B, C… indicate significant differences of Zn contents in the whole wheat flour for the same treatment among the different experimental fields (*p* ≤ 0.05).

**Table 3:** Grain Yield, Test Weight, Moisture Content and Thousand Kernel Weight (TKW) (*n* = 4) of *Triticum aestivum* L. grains, varieties Roxo and Paiva, from 1–4 experimental fields.

Experimental Field	Fertilizer	Variety	Treatment	Grain Yield (kg/ha)	Test Weight (kg/hl)	Moisture Content (%)	TKW (g)
**1**	**ZnSO_4_**	**Roxo**	**T0**	1970 ± 143 ^b,AB^	81.53 ± 0.15 ^ab,A^	10.63 ± 0.03 ^ab,B^	38.73 ± 0.45 ^a,AB^
			**T1**	1932 ± 58 ^b,A^	82.33 ± 1.10 ^ab,A^	10.38 ± 0.21 ^b,B^	34.30 ± 1.42 ^b,B^
			**T2**	1841 ± 82 ^b,A^	80.78 ± 0.40 ^b,A^	10.62 ± 0.08 ^ab,B^	33.39 ± 1.34 ^b,B^
		**Paiva**	**T0**	3379 ± 78 ^a,A^	83.35 ± 0.30 ^a,A^	10.66 ± 0.08 ^ab,B^	42.69 ± 0.46 ^a,AB^
			**T1**	3002 ± 256 ^a,A^	82.10 ± 0.30 ^ab,A^	10.91 ± 0.02 ^a,B^	41.09 ± 0.74 ^a,A^
			**T2**	3050 ± 117 ^a,A^	82.13 ± 0.30 ^ab,A^	10.87 ± 0.05 ^a,C^	41.67 ± 0.45 ^a,A^
**2**	**Zn-EDTA**	**Roxo**	**T0**	1691 ± 152 ^a,B^	81.78 ± 0.14 ^a,A^	10.34 ± 0.06 ^a,B^	39.43 ± 0.92 ^ab,AB^
			**T1**	1559 ± 217 ^a,A^	80.18 ± 0.84 ^a,AB^	10.28 ± 0.03 ^a,B^	40.05 ± 1.23 ^ab,A^
			**T2**	1621 ± 153 ^a,A^	79.35 ± 1.52 ^a,AB^	10.26 ± 0.04 ^a,B^	36.56 ± 1.41 ^b,AB^
		**Paiva**	**T0**	1572 ± 61 ^a,C^	80.38 ± 0.21 ^a,B^	10.29 ± 0.03 ^a,C^	41.08 ± 0.20 ^a,B^
			**T1**	1625 ± 211 ^a,B^	79.13 ± 0.54 ^a,B^	10.27 ± 0.02 ^a,C^	41.02 ± 0.53 ^a,A^
			**T2**	1472 ± 122 ^a,C^	78.80 ± 0.18 ^a,B^	10.17 ± 0.04 ^a,D^	39.31 ± 0.61 ^ab,AB^
**3**	**Tecnifol Zinc**	**Roxo**	**T0**	1689 ± 107 ^b,B^	76.95 ± 0.74 ^ab,B^	10.54 ± 0.04 ^bc,B^	28.68 ± 0.71 ^c,C^
			**T1**	1716 ± 64 ^b,A^	78.13 ± 0.33 ^a,B^	10.52 ± 0.03 ^bc,B^	30.04 ± 0.25 ^bc,C^
			**T2**	1594 ± 62 ^b,A^	76.95 ± 0.63 ^ab,B^	10.49 ± 0.09 ^c,B^	28.87 ± 0.85 ^c,C^
		**Paiva**	**T0**	2902 ± 123 ^a,AB^	74.58 ± 0.69 ^bc,C^	10.75 ± 0.04 ^ab,B^	34.76 ± 0.56 ^a,C^
			**T1**	2594 ± 232 ^a,A^	73.78 ± 0.70 ^c,C^	10.79 ± 0.06 ^a,B^	34.30 ± 1.91 ^ab,B^
			**T2**	2810 ± 157 ^a,A^	75.08 ± 0.05 ^bc,C^	10.68 ± 0.04 ^abc,C^	33.70 ± 0.66 ^ab,C^
**4**	**ZnSO_4_**	**Roxo**	**T0**	2349 ± 151 ^cdef,A^	82.7 ± 0.24 ^ab,A^	11.41 ± 0.19 ^bcd,A^	42.35 ± 0.68 ^ab,A^
			**T1**	1915 ± 113 ^fg,A^	82.10 ± 0.30 ^ab,A^	11.33 ± 0.13 ^cd,A^	39.97 ± 0.69 ^bcde,A^
			**T2**	1626 ± 142 ^g,A^	81.90 ± 0.36 ^ab,A^	11.58 ± 0.18 ^abcd,A^	38.46 ± 0.62 ^cdef,A^
		**Paiva**	**T0**	3180 ± 185 ^ab,A^	82.45 ± 0.44 ^ab,A^	12.00 ± 0.06 ^ab,A^	41.19 ± 0.60 ^abc,B^
			**T1**	3234 ± 109 ^a,A^	82.53 ± 0.23 ^ab,A^	11.91 ± 0.08 ^abcd,A^	42.47 ± 0.32 ^ab,A^
			**T2**	3047 ± 94 ^abc,A^	82.50 ± 0.29 ^ab,A^	11.70 ± 0.06 ^abcd,B^	37.78 ± 1.14 ^cdef,B^
	**Zn-EDTA**	**Roxo**	**T0**	1902 ± 6 ^fg,AB^	82.78 ± 0.19 ^ab,A^	11.49 ± 0.14 ^abcd,A^	36.61 ± 0.90 ^ef,B^
			**T1**	1799 ± 122 ^fg,A^	82.45 ± 0.36 ^ab,A^	11.29 ± 0.11 ^d,A^	35.25 ± 0.51 ^f,B^
			**T2**	1573 ± 44 ^g,A^	81.75 ± 0.21 ^b,A^	11.39 ± 0.04 ^bcd,A^	36.19 ± 0.76 ^f,AB^
		**Paiva**	**T0**	2452 ± 171 ^cdef,B^	82.98 ± 0.28 ^ab,A^	11.89 ± 0.09 ^abcd,A^	43.06 ± 0.67 ^ab,AB^
			**T1**	2495 ± 99 ^bcdef,A^	82.70 ± 0.16 ^ab,A^	11.66 ± 0.14 ^abcd,A^	42.23 ± 0.38 ^ab,A^
			**T2**	2203 ± 191 ^defg,B^	82.18 ± 0.28 ^ab,A^	11.88 ± 0.08 ^abcd,AB^	40.81 ± 0.66 ^bcd,AB^
	**Tecnifol Zinc**	**Roxo**	**T0**	2077 ± 122 ^efg,AB^	82.73 ± 0.20 ^ab,A^	11.64 ± 0.13 ^abcd,A^	35.66 ± 1.21 ^f,B^
			**T1**	1951 ± 168 ^fg,A^	81.90 ± 0.17 ^ab,A^	11.62 ± 0.15 ^abcd,A^	37.46 ± 0.77 ^def,AB^
			**T2**	1831 ± 155 ^fg,A^	82.40 ± 0.10 ^ab,A^	11.44 ± 0.14 ^abcd,A^	36.82 ± 0.28 ^ef,AB^
		**Paiva**	**T0**	2827 ± 217 ^abcd,AB^	83.30 ± 0.33 ^a,A^	11.77 ± 0.13 ^abcd,A^	44.52 ± 0.13 ^a,A^
			**T1**	2731 ± 125 ^abcde,A^	82.88 ± 0.31 ^ab,A^	11.93 ± 0.15 ^abc,A^	42.96 ± 0.31 ^ab,A^
			**T2**	2808 ± 65 ^abcd,A^	82.80 ± 0.21 ^ab,A^	12.07 ± 0.03 ^a,A^	42.20 ± 0.56 ^ab,A^

For the same fertilizer (ZnSO_4_, Zn-EDTA and Tecnifol Zinc): T0 = control; T1, T2 correspond, respectively, to initial and upper concentration. Letters a, b, c… indicate, within each item, significant differences among treatments, for both varieties in the same experimental field and letters A, B, C… indicate, within each item, significant differences among each treatment, for each variety and among all experimental fields (*p* ≤ 0.05).

**Table 4:** Ash Content (*n* = 3) and Colorimeter parameters—CIELab System (*n* = 12) of whole wheat flour of *Triticum aestivum* L., varieties Roxo and Paiva, from 1–4 experimental fields.

Experimental Field	Fertilizer	Variety	Treatment	Ash Content (%)	Colorimeter—CIELab System		
					L*	a*	b*
**1**	**ZnSO_4_**	**Roxo**	**T0**	1.839 ± 0.030 ^bc,B^	81.67 ± 0.610 ^a,AB^	−1.450 ± 0.170 ^bc,AB^	22.48 ± 0.370 ^ab,AB^
			**T1**	1.872 ± 0.013 ^b,A^	82.33 ± 0.340 ^a,A^	−1.900 ± 0.070 ^c,BC^	22.45 ± 0.270 ^ab,AB^
			**T2**	1.833 ± 0.055 ^bc,AB^	82.61 ± 0.460 ^a,A^	−1.950 ± 0.090 ^c,BC^	21.97 ± 0.250 ^ab,AB^
		**Paiva**	**T0**	3.234 ± 0.108 ^a,A^	77.55 ± 1.670 ^b,A^	−1.190 ± 0.190 ^ab,B^	20.90 ± 0.630 ^b,A^
			**T1**	1.589 ± 0.020 ^cd,BC^	77.99 ± 0.520 ^b,AB^	−0.760 ± 0.190 ^a,AB^	22.77 ± 0.460 ^a,A^
			**T2**	1.534 ± 0.052 ^d,B^	79.48 ± 0.580 ^ab,A^	−1.370 ± 0.110 ^bc,B^	21.23 ± 0.140 ^ab,AB^
**2**	**Zn-EDTA**	**Roxo**	**T0**	1.480 ± 0.030 ^a,D^	81.61 ± 0.390 ^a,AB^	−2.040 ± 0.040 ^b,C^	21.15 ± 0.190 ^a,B^
			**T1**	1.600 ± 0.046 ^a,C^	82.33 ± 1.090 ^a,A^	−2.210 ± 0.160 ^b,C^	20.66 ± 0.420 ^a,C^
			**T2**	1.558 ± 0.052 ^a,C^	82.91 ± 0.730 ^a,A^	−2.130 ± 0.180 ^b,C^	21.80 ± 0.480 ^a,B^
		**Paiva**	**T0**	1.648 ± 0.036 ^a,BC^	72.30 ± 0.430 ^b,B^	−0.780 ± 0.070 ^a,AB^	20.77 ± 0.190 ^a,A^
			**T1**	1.522 ± 0.034 ^a,C^	75.91 ± 1.190 ^b,BC^	−1.000 ± 0.110 ^a,AB^	21.09 ± 0.130 ^a,B^
			**T2**	1.576 ± 0.030 ^a,B^	74.59 ± 1.300 ^b,B^	−0.820 ± 0.160 ^a,A^	20.94 ± 0.150 ^a,B^
**3**	**Tecnifol Zinc**	**Roxo**	**T0**	2.024 ± 0.040 ^a,A^	79.30 ± 0.400 ^a,B^	−1.080 ± 0.130 ^bc,AB^	23.22 ± 0.330 ^a,A^
			**T1**	1.811 ± 0.018 ^b,A^	77.87 ± 0.450 ^ab,B^	−1.590 ± 0.090 ^c,AB^	21.71 ± 0.170 ^b,BC^
			**T2**	1.814 ± 0.023 ^b,B^	79.32 ± 0.900 ^a,B^	−1.470 ± 0.080 ^c,A^	22.13 ± 0.160 ^b,AB^
		**Paiva**	**T0**	1.785 ± 0.027 ^b,BC^	76.09 ± 0.620 ^bc,A^	−0.760 ± 0.170 ^ab,AB^	21.44 ± 0.390 ^b,A^
			**T1**	1.786 ± 0.039 ^b,A^	73.48 ± 0.860 ^c,C^	−0.530 ± 0.130 ^a,A^	21.83 ± 0.190 ^b,AB^
			**T2**	1.833 ± 0.008 ^b,A^	77.93 ± 0.650 ^ab,A^	−0.910 ± 0.160 ^ab,AB^	21.79 ± 0.190 ^b,A^
**4**	**ZnSO_4_**	**Roxo**	**T0**	1.982 ± 0.011 ^a,A^	80.88 ± 0.240 ^abc,AB^	−1.680 ± 0.130 ^ij,BC^	21.86 ± 0.350 ^bcdef,B^
			**T1**	1.890 ± 0.006 ^ab,A^	80.39 ± 0.490 ^abcde,A^	−1.400 ± 0.070 ^defghi,A^	23.24 ± 0.180 ^a,A^
			**T2**	1.999 ± 0.032 ^a,A^	80.81 ± 0.540 ^abcd,AB^	−1.460 ± 0.140 ^efghi,A^	23.15 ± 0.380 ^ab,A^
		**Paiva**	**T0**	1.846 ± 0.007 ^bc,B^	76.43 ± 0.280 ^g,A^	−0.630 ± 0.090 ^a,A^	21.94 ± 0.370 ^abcde,A^
			**T1**	1.737 ± 0.023 ^cde,A^	79.48 ± 0.540 ^bcdef,A^	−1.160 ± 0.060 ^bcdef,B^	21.08 ± 0.070 ^ef,B^
			**T2**	1.782 ± 0.027 ^bcd,A^	78.03 ± 0.290 ^efg,A^	−1.180 ± 0.100 ^bcdefg,AB^	21.08 ± 0.340 ^ef,AB^
	**Zn-EDTA**	**Roxo**	**T0**	1.710 ± 0.009 ^def,C^	79.65 ± 1.190 ^bcdef,B^	−1.570 ± 0.100 ^ghij,BC^	22.10 ± 0.440 ^abcde,AB^
			**T1**	1.762 ± 0.048 ^bcde,AB^	79.88 ± 0.420 ^abcdef,AB^	−1.560 ± 0.070 ^fghij,AB^	22.44 ± 0.290 ^abcd,AB^
			**T2**	1.745 ± 0.030 ^cde,B^	80.77 ±0.370 ^abcd,AB^	−1.880 ± 0.030 ^hij,ABC^	21.66 ± 0.310 ^cdef,B^
		**Paiva**	**T0**	1.631 ± 0.032 ^efgh,BC^	77.65 ±0.480 ^fg,A^	−0.650 ± 0.080 ^a,A^	21.56 ± 0.270 ^cdef,A^
			**T1**	1.686 ± 0.024 ^defg,AB^	78.15 ±0.610 ^defg,AB^	−0.900 ± 0.040 ^abc,AB^	21.46 ± 0.140 ^def,B^
			**T2**	1.529 ± 0.025 ^h,B^	79.30 ± 0.240 ^bcdef,A^	−1.230 ± 0.020 ^cdefgh,AB^	20.59 ± 0.080 ^f,B^
	**Tecnifol Zinc**	**Roxo**	**T0**	1.592 ± 0.020 ^fgh,CD^	82.40 ± 0.520 ^a,A^	−1.810 ± 0.070 ^ij,BC^	22.11 ± 0.140 ^abcde,AB^
			**T1**	1.648 ± 0.018 ^defgh,BC^	81.63 ± 0.470 ^abc,A^	−1.770 ± 0.080 ^ij,AB^	22.49 ± 0.280 ^abcd,AB^
			**T2**	1.707 ± 0.024 ^defg,BC^	81.97 ± 0.360 ^ab,A^	−1.610 ± 0.090 ^j,AC^	22.82 ± 0.140 ^abc,AB^
		**Paiva**	**T0**	1.572 ± 0.028 ^gh,C^	77.53 ± 0.720 ^fg,A^	−0.780 ± 0.060 ^ab,AB^	21.42 ± 0.160 ^def,A^
			**T1**	1.522 ± 0.027 ^h,C^	77.81 ±0.700 ^efg,AB^	−1.030 ± 0.080 ^abcd,B^	21.39 ± 0.240 ^def,B^
			**T2**	1.529 ± 0.028 ^h,B^	79.08 ± 0.440 ^cdefg,A^	−1.070 ± 0.040 ^bcde,AB^	21.84 ± 0.210 ^bcdef,A^

For the same fertilizer (ZnSO_4_, Zn-EDTA and Tecnifol Zinc): T0 = control; T1, T2 correspond, respectively, to initial and upper concentration. Letters a, b, c… indicate, within each item, significant differences among treatments, for both varieties in the same experimental field and letters A, B, C… indicate, within each item, significant differences among each treatment, for each variety and among all experimental fields (*p* ≤ 0.05). Color parameters: L*—lightness; a*—red–green transitions; b*—yellow–blue transitions.

## Data Availability

Not applicable.
